# Suction, flexion, and noise profile of three flexible and navigable suction ureteral access sheaths: a benchtop comparison

**DOI:** 10.1007/s00345-026-06253-z

**Published:** 2026-02-20

**Authors:** Connor A. Church, Elijah Haynal, Ye Jean Lim, Ye Na Diana Lim, Frankis Almaguel, Elizabeth A. Baldwin, Ruben Crew, Matthew Buell, Ali Albaghli, D. Duane Baldwin

**Affiliations:** Department of Urology, Loma Linda University Health, 11234 Anderson Street, Loma Linda, CA 92354 USA

**Keywords:** Ureteroscopy, Kidney stones, Ureteral access sheath, Urolithiasis, Retrograde intrarenal surgery

## Abstract

**Purpose:**

Flexible and navigable suction ureteral access sheaths (FANS) have revolutionized treatment of urinary stones, and we hypothesized that different sheaths may have different functional characteristics. The purpose of this study was to compare 3 different FANS in a benchtop kidney/ureter model.

**Methods:**

Three 12/14 Fr FANS were compared in a 3D-printed silicon kidney/ureter model: Dornier Hoover, Wellead ClearPetra, and Vathin. Randomized stone-suction trials were done in the upper, interpolar, and lower poles. Maximum deflection using 6.3 and 7.5 Fr flexible ureteroscopes, deflection control force, suction flow rates, and noise levels were also compared.

**Results:**

Hoover and ClearPetra FANS suctioned more fragments than Vathin when all kidney trials were considered (*p* < 0.01 for all). Comparing deflection, Hoover and Vathin had greater downward deflection compared to ClearPetra for both ureteroscopes (*p* < 0.005). Hoover upward deflection exceeded ClearPetra for both ureteroscopes (*p* < 0.008) and Vathin using the 6.3 Fr ureteroscope (*p* < 0.001). Deflection control force was less for Hoover and Vathin than ClearPetra at all angles in both directions (*p* < 0.001). Suction flow rates were higher for Hoover and ClearPetra compared to Vathin. At maximum deflection, ClearPetra flow rate exceeded Hoover (*p* < 0.001). ClearPetra was quietest during suction (54.5dB), while Vathin was loudest (74.0dB) (*p* < 0.001).

**Conclusion:**

When comparing all kidney trials, Hoover and ClearPetra FANS outperformed Vathin in stone suction. Hoover and Vathin showed greater deflection and lower deflection force compared to ClearPetra. ClearPetra had higher flow rates and was quieter.

## Introduction

Kidney stones affect around 9.9% of the adult U.S. population, with prevalence steadily increasing worldwide [1]. Stone burden contributes an estimated healthcare cost of more than $5 billion [2] each year and may acutely result in pain, pyelonephritis, hydronephrosis, and sepsis [3] while also being a risk factor for chronic conditions such as kidney failure, chronic kidney disease, diabetes, and cardiovascular disease [1]. Retrograde Intrarenal Surgery (RIRS) is one of the most commonly indicated methods for stone removal, being recommended as first-line treatment for stones under 2 cm in diameter [4]. Due to its non-invasive nature, RIRS normally yields fewer complications than Percutaneous Nephrolithotomy (PCNL), which is typically used to clear stones larger than 2 cm and staghorn calculi, although evidence exists showing RIRS as a safe but slightly less efficacious alternative for large stone removal [5].

The introduction of the ureteral access sheath (UAS) aided RIRS by allowing for repeated entry of the ureteroscope, while also improving irrigation outflow and lowering intrarenal pressure to prevent pyelovenous backflow during surgery [6]. More recently, flexible and navigable suction ureteral access sheaths (FANS) have been demonstrated to be superior to traditional UAS in several areas, with meta-analyses showing a significant increase in stone-free rate (SFR), a reduction in surgical complications and postoperative fever events, and a decrease in the average length of hospital stay [7].

Several facets of this new technology remain unexplored, including the deflectability with new 6.3 Fr disposable ureteroscopes (compared to 7.5 Fr), the force required for deflection with different FANS, and the noise generated during use. In addition, the novel Vathin FANS has not been compared to other FANS. We hypothesized that different FANS sheaths may have different functional characteristics. The purpose of this benchtop study was to compare the suction efficiency, fragment evacuation, deflection, deflection control force, and noise generation between three different FANS in simulated RIRS.

## Methods

The three FANS evaluated were the Dornier Hoover Negative Pressure UAS 50 cm (Dornier MedTech, Weßling, Germany), Wellead Medical ClearPetra^®^ UAS 46 cm (Well Lead Medical Co., Guangzhou, China), and Vathin Single-use UAS 45 cm (Hunan Vathin Medical Instrument Co., Hunan, China).

### Model creation

Silicone kidney models were created using de-identified patient CT scans. 3D slicer (Kitware, New York, USA) was used to create molds of the kidney and collecting system, which were 3D printed by an Ultimaker 3 Extended 3D printer (Ultimaker, Utrecht, Netherlands) using polyactic acid (3D Universe, Chicago, IL). The mold was then filled with Dragon Skin™ 20 (Smooth-On, Inc., Macungie, PA) and allowed to fully cure. Three models were created. In each model, a medical-grade thermoplastic tube was inserted into either the upper, interpolar, or lower pole to allow for the introduction of stone fragments. Following introduction of the stone fragments, the kidney model was sealed using Parafilm (Uline, Pleasant Prairie, WI).

Ureteral models were created by 3D printing a mold of the inner lumen of the ureter and covering it with silicone. Autodesk Fusion 360 (Autodesk, San Francisco, CA) was used to design the inner lumen 3D model using physiologically accurate measurements [8–10], and Dragon Skin™20 was used to coat the model. The ureter was then secured to the kidney using Parafilm to create a watertight seal.

### Stone creation

**Fig. 1:**
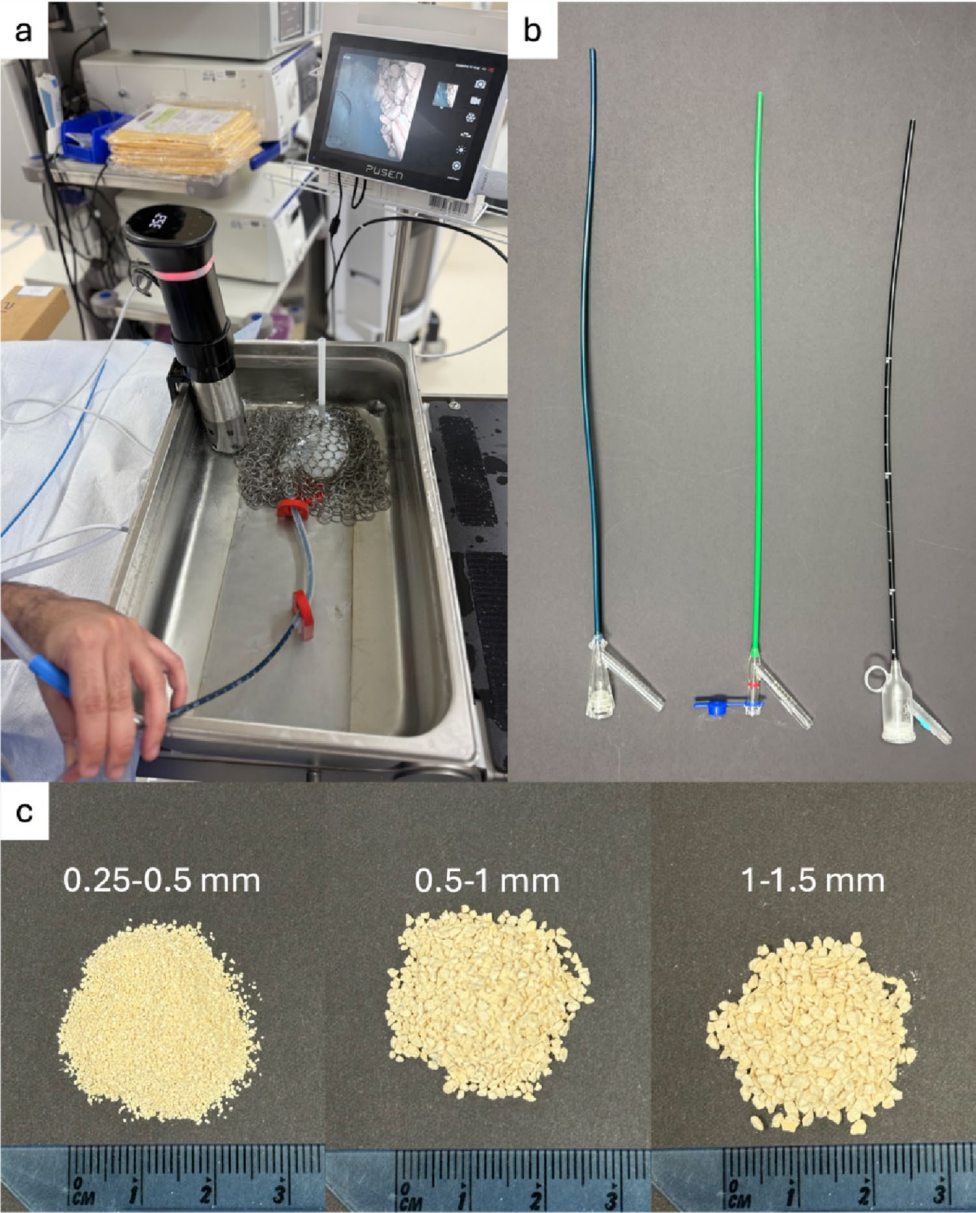
**a** Stone-fragment suction setup, **b** The three FANS from left to right: Hoover, ClearPetra, and Vathin, **c** 1 g samples of the different stone-fragment sizes used for suction trials

Artificial kidney stones were created using a 15:3 BegoStone (Bego USA, Lincoln, RI) to water ratio to simulate calcium oxalate monohydrate kidney stones [11]. After drying, the stones were crushed into small fragments and sieves were used to collect different stone sizes (0.25–0.5 mm, 0.5–1.0 mm, 1.0–1.5 mm) (Fig. 1).

### Study trials–stone evacuation

Stone fragment suction trials were performed by placing the synthetic kidney model into a scaffold mimicking the natural course of the ureter and submerging the setup in a 18 × 28’’ stainless steel basin filled with water at 37° C (Fig. 1). Prior to each trial, a FANS paired with a Pusen 7.5 Fr disposable FURS was advanced into the pole being evaluated. One gram of dry stone-fragments was then inserted, and the fragments were suctioned for 3 min. Pressure-bag irrigation at 250 mmHg and continuous suction at 480 mmHg from a Neptune 3 (Stryker Corporation, Kalamazoo, MI) suction device were used, as these are the settings routinely employed at our institution. With the ureteroscope at the tip of the sheath, the sheath was navigated until the stone fragments were identified. The ureteroscope was then removed with suction to evacuate stone fragments. Fragments were collected using a stone-collection bottle with a fine screen after irrigation of stone fragments from the tubing. After each trial, the collected fragments were removed, dried in a warm, dry room for 48 h, and weighed to determine the amount of fragments suctioned. Five trials, randomized for sheath size and renal location were performed for each stone size range.

### Study trials–suction rate

**Fig. 2:**
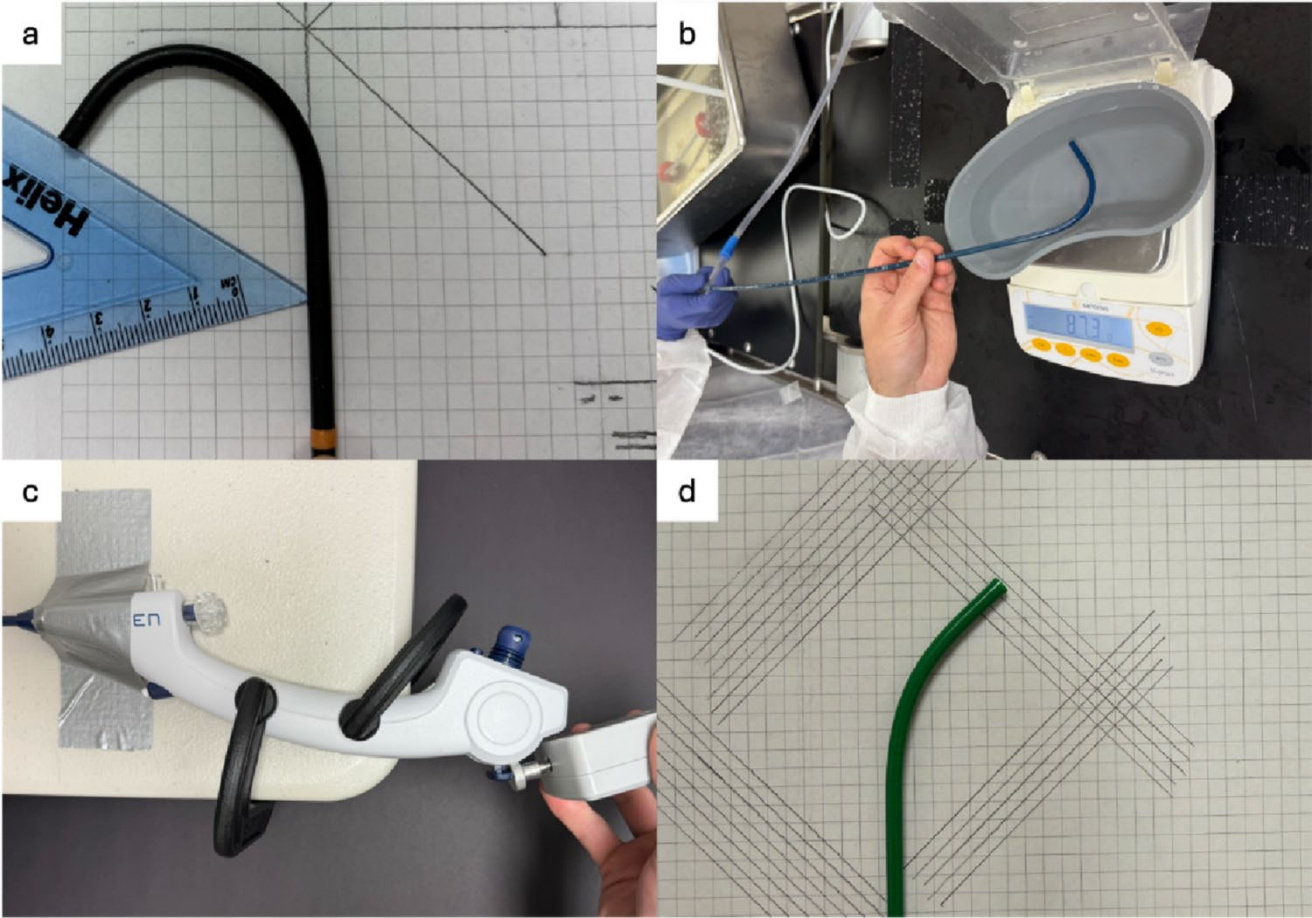
**a** Example measurement for maximum angle of deflection, **b** Setup for suction to measure liquid flow rate, **c** Deflection control force setup at the clamped handle, **d** Deflection control force setup at the tip

To evaluate the flow rate of liquid suction, a graduated plastic basin was placed on a high-sensitivity scale and filled with 300 mL of water (Fig. 2). The FANS was submerged in the water with continuous suction at 480 mmHg. The time taken to suction 100 mL was recorded for each trial. Trials were performed at 0°, 90°, and at maximum deflection using a Pusen 7.5 Fr disposable FURS with the tip positioned at the tip of the FANS with the vent closed. An additional trial was performed with the FURS retracted to the bifurcation of the sheath suction port. The angle of the sheath was maintained constant in relation to the ground. Time taken to suction was then converted to flow rate (cc/second).

### Study trials–deflection

To measure the maximum angle of deflection, each FANS was paired with a 7.5 Fr Pusen disposable FURS and a 6.3 Fr Hugemed disposable FURS and flexed to its maximum angle of deflection with the tip of the ureteroscope at the tip of the FANS (Fig. 2). The 12/14Fr FANS/7.5Fr disposable flexible ureteroscope combination was selected as this combination has been shown to result in the fastest stone evacuation [12]. The 6.3Fr disposable flexible ureteroscope was selected to test deflection with a smaller caliber ureteroscope. The force required to deflect the 7.5 Fr ureteroscope to 45°, 90°, and 135° was measured using a HOJILA electronic force gauge (Hangzhou Shengyi Health Management Co., Hangzhou, China) (Fig. 2).

### Study trials–noise

A setup similar to that used for liquid suction measurement was used to measure noise levels. A 7.5 Fr Pusen disposable FURS was paired with each sheath for each trial with no flexion engaged, and a suction device (480 mmHg) was used to suction water. For each trial, complete suction was engaged at the FANS handle for 10 s followed by a 10-second period of passive suction where the surgeon’s finger was not covering the suction valve or the sliding valve was kept open. An Extech HD600 decibel meter (Extech Instruments, Nashua, New Hampshire) 20 cm away from the FANS recorded noise levels.

### Equipment specifications

There were notable differences in measured specifications among the three devices. For each FANS, the narrowest point of the lumen was at the flexible tip (11.82 Fr, 12.18 Fr, and 11.70 Fr for the Hoover, ClearPetra, and Vathin). The length of the flexible portion was 102.7 mm for the Hoover, 96.1 mm for the ClearPetra, and 76.0 mm for the Vathin. The distal tip of the FANS consisted of a 1 mm soft flexible tip for the Hoover, a 4 mm soft flexible tip for the ClearPetra, and a 6 mm solid metal ring with no soft distal portion for the Vathin.

### Statistical analysis

Statistical analysis was performed using Jamovi version 2.7.6 (The Jamovi Project, Sydney, Australia). One-way ANOVA with parametric post-hoc analysis was used for flow rate, deflection angle, and deflection control force as this data exhibited normal distribution. Kruskal-Wallis analysis was used for noise levels as the data was not normally distributed. Linear regression was used for stone suction. Pair-wise comparisons were evaluated using a Bonferroni correction for multiple testing (α = 0.0167).

## Results

The Hoover (0.569 ± 0.246 g) and ClearPetra (0.558 ± 0.268 g) FANS suctioned more fragments than the Vathin (0.426 ± 0.301 g) when all trials were compared throughout the kidney (*p* < 0.01 for all).

**Fig. 3:**
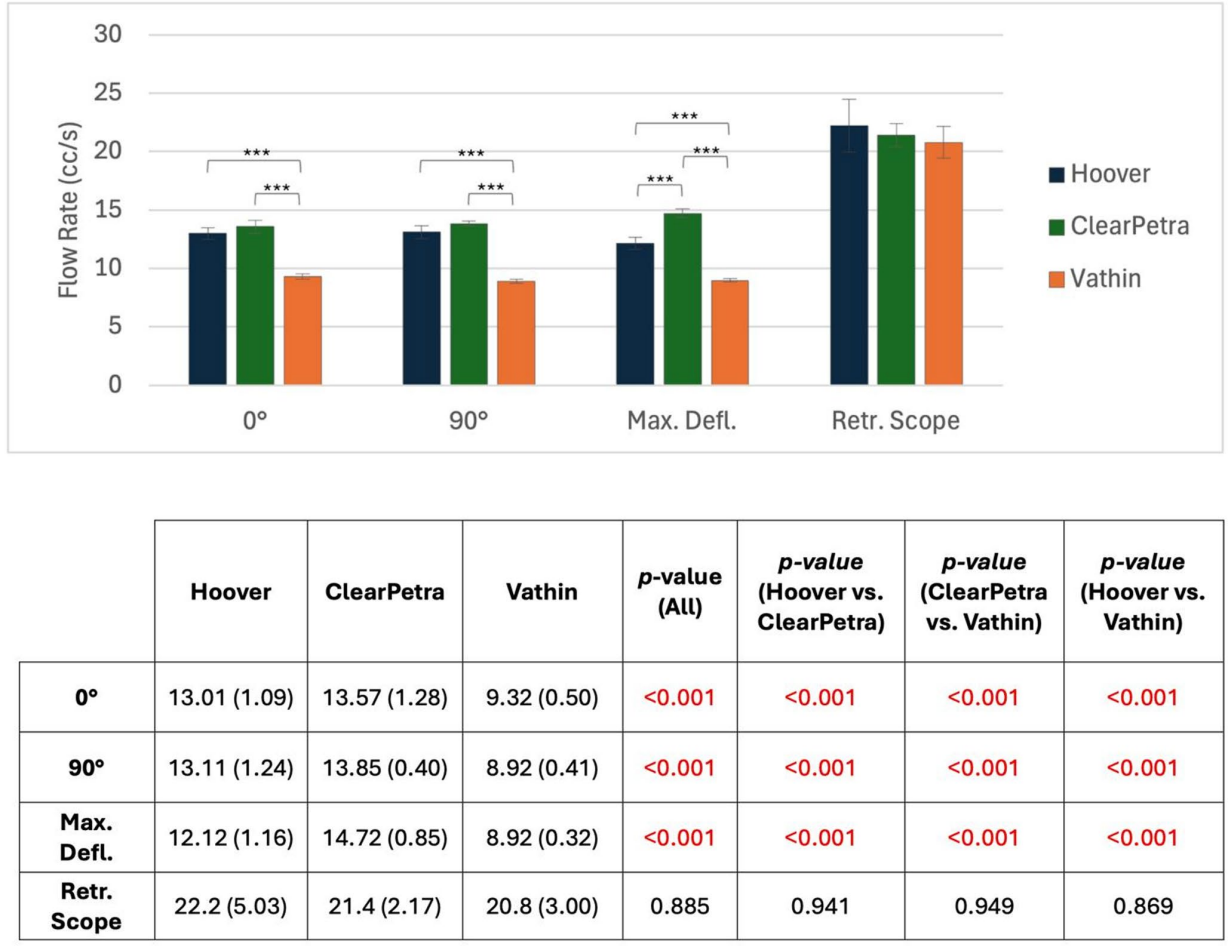
Mean liquid flow rate at 480mmHg suction for each FANS. * = *p* < 0.0167, ** = *p* < 0.01, *** = *p* < 0.001

At all angles with the FURS inserted to the tip of the FANS (0°, 90°, and maximum deflection), the Hoover and ClearPetra FANS exhibited significantly higher flow rates than the Vathin (*p* < 0.001 for all; Fig. 3). At the maximum achievable deflection angle, the ClearPetra (14.72 ± 0.85 cc/s) also had a significantly higher flow rate than the Hoover (12.12 ± 1.16 cc/s) (*p* < 0.001). When the ureteroscope was retracted to the suction port bifurcation, there was no difference in suction rates between the three FANS.

**Fig. 4:**
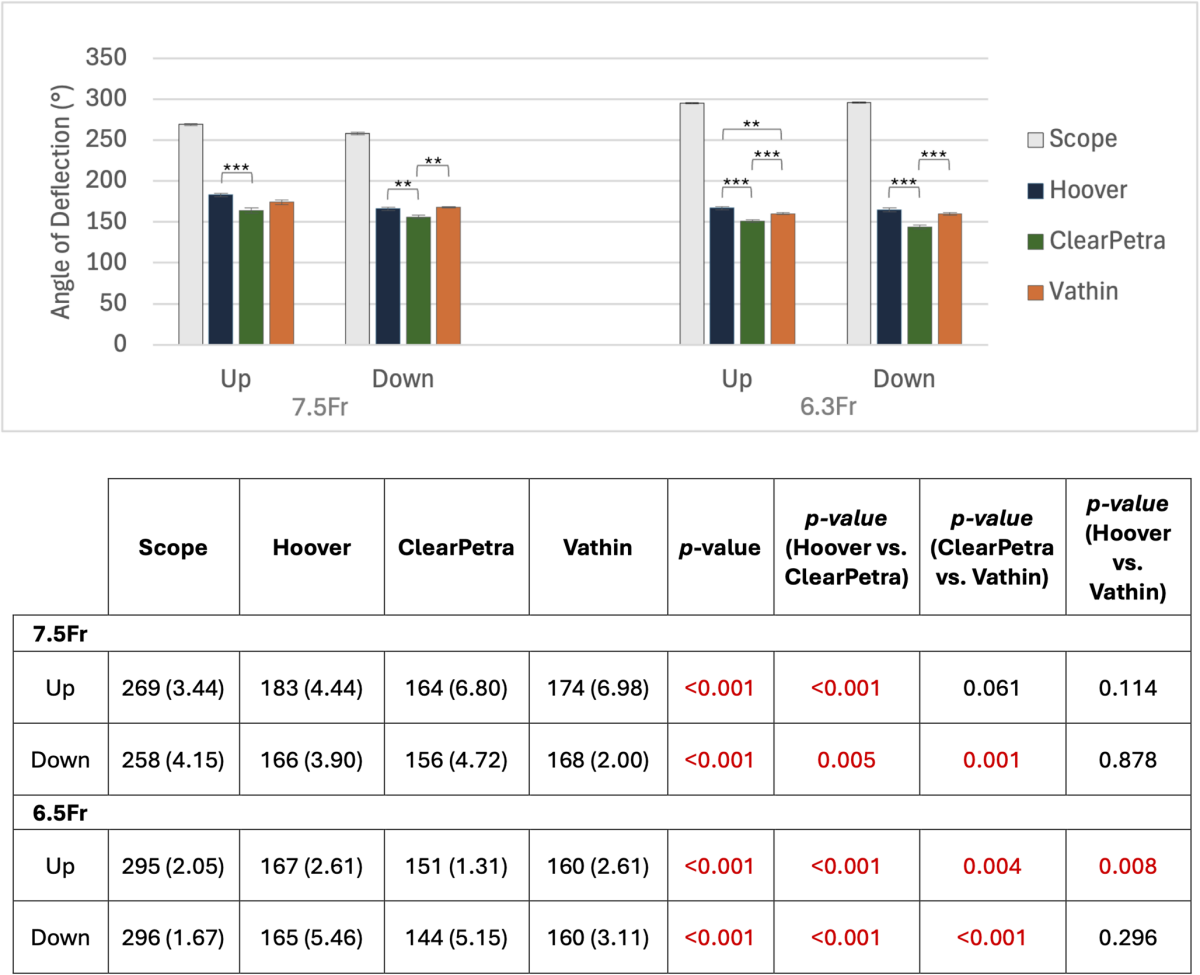
Mean deflection control force required to deflect each FANS to 45**°**, 90°, and 135°. All comparisons against the scope alone were significant (*p* < 0.001 for all). * = *p* < 0.0167, ** = *p* < 0.01, *** = *p* < 0.001

Using the 7.5 Fr ureteroscope, the Hoover (166 ± 3.90°) and Vathin (168 ± 2.00°) FANS exhibited significantly larger maximum angles of deflection when flexing down compared to the ClearPetra (156 ± 4.72°) (*p* ≤ 0.01 for both; Fig. 4) with no significant differences being observed between the Hoover and Vathin. When flexing up, only the maximum deflection of the Hoover (183 ± 4.44°) was significantly greater than the ClearPetra (164 ± 6.80°) (*p* < 0.001).

Using the 6.3 Fr ureteroscope, the maximum deflection downward of the Hoover (165 ± 5.46°) and Vathin (160 ± 3.11°) FANS were significantly greater than that of the ClearPetra (144 ± 5.15°) (*p* < 0.001 for both), with no significant differences between the Hoover and Vathin. The maximum deflection upward of the Hoover (167 ± 2.61°) was significantly greater than both the Vathin (160 ± 3.11°) and the ClearPetra (144 ± 5.15°) (*p* ≤ 0.01 for both), though the angle of the Vathin was still significantly greater than that of the ClearPetra (*p* < 0.001).

**Fig. 5:**
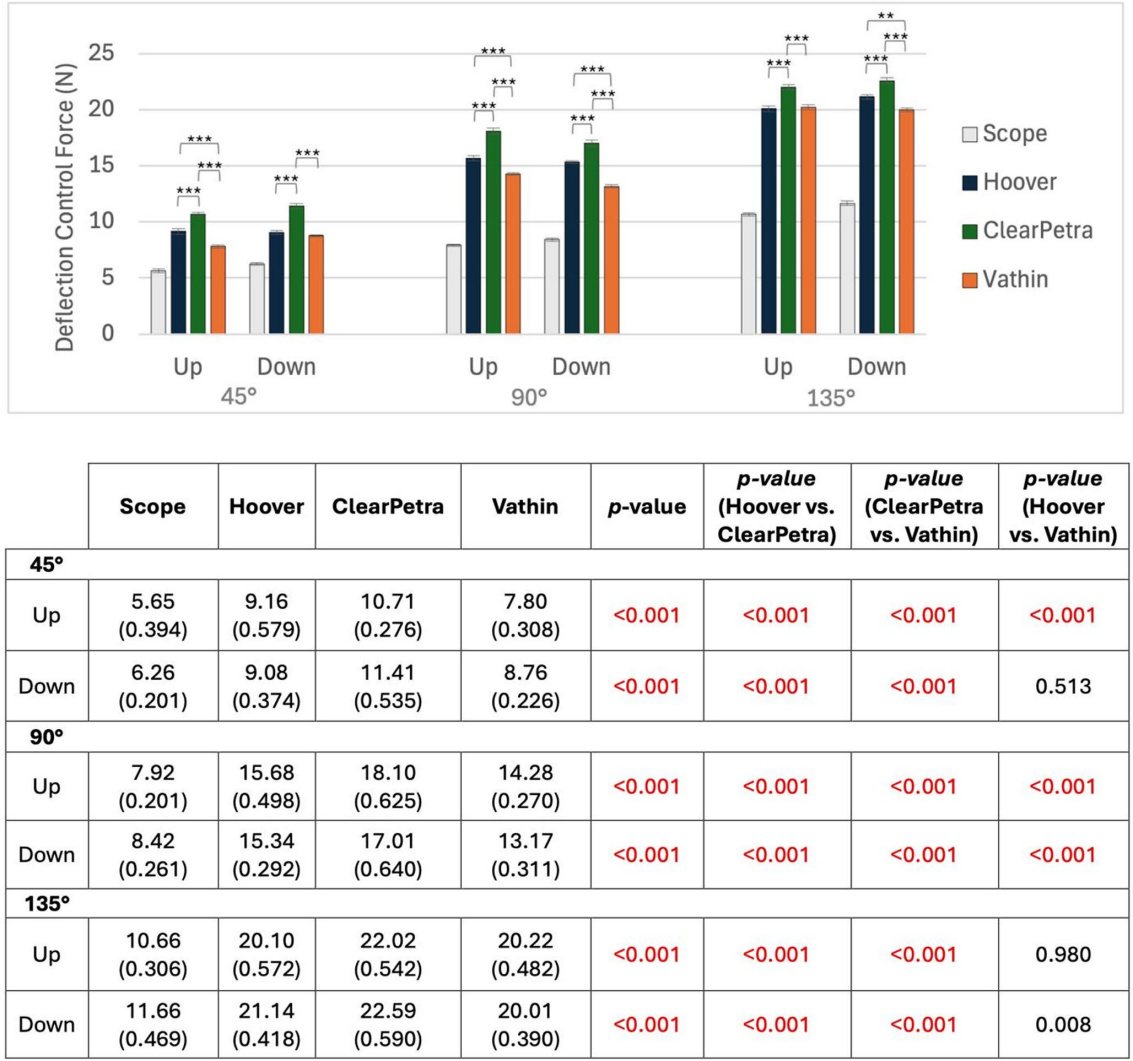
Mean deflection control force required to deflect each FANS to 45**°, **90°, and 135°. All comparisons against the scope alone were significant (*p* < 0.001 for all). * = *p* <0.0167, ** = *p* <0.01, *** = *p* <0.001

At every angle (45°, 90°, 135°) and in both directions, the Hoover and Vathin FANS required significantly less force to deflect than the ClearPetra (*p* < 0.001 for all; Fig. 5). The Vathin required significantly less force than the Hoover at 45° flexing up (*p* < 0.001), 90° flexing up and down (*p* < 0.001 for both), and 135° flexing down (*p* < 0.01).

With suction actively engaged, the ClearPetra FANS (54.5 ± 1.32 dB) was significantly quieter than both the Hoover (64.4 ± 0.715 dB) and the Vathin (74.0 ± 1.12 dB) (*p* < 0.001 for both). The Hoover was quieter than the Vathin (*p* < 0.001). With passive suction, the Vathin (66.1 ± 0.786 dB) and Hoover (66.4 ± 1.34 dB) FANS were significantly quieter than the ClearPetra (68.3 ± 0.720 dB) (*p* < 0.001 for both).

## Discussion

Improved SFR post-ureteroscopy reduces future stone events, re-interventions, and perioperative complications [13, 14]. Meta-analyses have shown FANS to increase SFR while reducing operative times and complications such as bleeding, infection, and sepsis when compared to traditional UAS [15]. Active suction with FANS also maintains lower intrarenal pressure compared to traditional non-suctioning sheaths, preventing pyelovenous backflow [15]. FANS also offer advantages when compared to other novel technology. When compared to Direct In-scope Suction (DISS), FANS were found to have comparable SFR with reduced operating times [16]. Compared to mini-PCNL, FANS displayed comparable SFR and reduced complication rates, though mini-PCNL operating times were shorter [17, 18]. Overall, studies have demonstrated FANS to improve intra-operative complications and post-operative outcomes. Given the increasing number of FANS on the market, understanding the characteristics of each sheath will enable scenario-indicated sheath selection.

Our study found the Hoover and ClearPetra FANS to suction more stones than the Vathin, clearing 33.6% and 31.0% more fragments, respectively. Differences in suction efficiency may be attributed to the unique qualities of each sheath. The Vathin FANS incorporates novel “snake-bone” technology in order to increase sheath flexibility. This new snake-bone arrangement of metal scales in the flexible portion sits in contrast to the coiled wire design of the Hoover and ClearPetra FANS.

When examining sheath flexibility, we found the Hoover and Vathin FANS to flex more and require less force to deflect when compared to the ClearPetra. While the performance of the Vathin appears to support the novel snake-bone design, we found that the ureteroscope was sometimes caught on these exposed scales, reducing the ability to slide the sheath over the ureteroscope. Differences in flexion did not appear to translate directly to an increase in stone removal in our kidney model trials. Still, advances in flexion may lead to increased surgeon comfort and control and may reduce fatigue during long procedures.

Flow rates in our study were highest for the ClearPetra and Hoover FANS when compared to the Vathin. The ClearPetra outperformed the Hoover at maximum deflection, which may be considered a decent proxy for accessing the lower pole. It is important to note, however, that the maximum angle of deflection was significantly larger for the Hoover when compared to the ClearPetra. This additional flow rate may in part compensate for the ClearPetra’s inability to flex to the level of the Hoover and Vathin when used for stone suction. The snake-bones of the Vathin FANS may contribute to turbulent flow during suction, potentially providing a reason for its decreased flow rates when compared to the other sheaths.

Our study found the average noise level of the Vathin FANS (74.0 dB) during active suction to be 157.7% higher than that of the Hoover (64.4 dB) and 196.0% higher than the ClearPetra (54.5 dB). Noise levels appeared to decrease with decreased proximal hub size, as less turbulence was observed in the ClearPetra (8.7 mm) capsule when compared to the larger Hoover (15.2 mm) and Vathin (22.5 mm) capsules. Elevated noise levels during urologic surgery have been shown to increase errors in communication, with one study showing average levels as low as 78.79 dB to cause a significant number of errors [19]. Average levels of 81.8 dB were shown to be standard in the operating room, a value similar to that of a train 30 feet away [19]. It is recommended to keep noise levels below 85–90 dB, as prolonged exposure (over 8 h) may lead to hearing damage and tinnitus [20, 21]. While none of the noise levels in our study appear to breach dangerous noise thresholds, elevated levels in the operating room may still cause irritation and impair communication.

Our study is limited by the use of a benchtop silicone model that does not completely recreate the exact distensibility of the human kidney and the frictional properties and physical characteristics of an actual patient. However, use of this benchtop model allowed us to reproduce trials in a standardized fashion. In addition, we did not compare every FANS on the market. Furthermore, the FANS used in the study did not come in exactly the same lengths, so the most similar lengths possible were employed (45 cm Vathin, 46 cm ClearPetra, 50 cm Hoover). It is possible that these differences in length could result in differences in flow dynamics and potentially contribute to differences in noise generation. In addition, only one diameter and length of sheath was tested. It is possible that results could vary with different sheath and ureteroscope dimensions. A final limitation of this study is that intrarenal pressures were not monitored due to the fact that the silicon material that the kidney was made of did not have the same distensibility as a renal collecting system. The effect of these sheaths upon intrarenal pressure could be compared in future animal or clinical series. Despite these limitations, this is the first comprehensive head-to-head comparison between these three novel FANS devices.

## Conclusion

When comparing all trials throughout the kidney, the Hoover and ClearPetra FANS outperformed the Vathin in stone suction. The Hoover and Vathin showed greater maximum angle of deflection and lower deflection forces compared to the ClearPetra, while the ClearPetra had the highest flow rates and was quietest.

## Data Availability

Data supporting the findings of this study are available upon request from the corresponding author.
